# Analyzing the Content Found on Fellowship Websites for Adult Congenital Heart Disease

**DOI:** 10.7759/cureus.42682

**Published:** 2023-07-30

**Authors:** Wesam Almasri, Mahfujul Z Haque, Moaid Shaik, Abdul Mannan, Sheema Rehman, Mashkur Husain

**Affiliations:** 1 Medicine, Oakland University William Beaumont School of Medicine, Northville, USA; 2 Medicine, Michigan State University College of Human Medicine, East Lansing, USA; 3 Medicine, Michigan State University College of Osteopathic Medicine, East Lansing, USA; 4 Internal Medicine, University at Buffalo, Buffalo, USA; 5 Internal Medicine, Henry Ford Health System, Detroit, USA; 6 Interventional Cardiology, Henry Ford Health System, Wynadotte, USA

**Keywords:** education, accessibility, fellowship, adult congenital heart disease, cardiology

## Abstract

The Adult Congenital Heart Disease (ACHD) fellowship is a two-year fellowship that can be done by physicians who have finished their internal medicine residency and cardiology fellowship. This study evaluated the accessibility and provided information on the websites of the ACHD fellowship programs to identify potential areas of improvement for future fellowship applicants. Analysis of 25 ACHD fellowship program websites was conducted based on 34 criteria under three main categories: recruitment information, education and research information, and incentive information. This study found that many evaluated ACHD program websites lacked information regarding recruitment. Specifically, information regarding mentorship opportunities, hospital statistics/number of beds, selection process, and interview dates, leaving out crucial details on what to expect during the matching process. Additionally, more information on education and research is beneficial for applicants to sufficiently compare ACHD fellowship programs and make more informed decisions about which programs they would like to apply to. Information on academic stipends, evaluation criteria, expected caseload, moonlighting opportunities, elective opportunities, rotation schedules, call requirements, and types of procedures were all limited across multiple websites. Lastly, incentive information was found to be insufficient across most ACHD fellowship websites. Incentive information included fellow wellness, harassment policies, parental leave, salary, benefits, and vacation/sick leave. This study shows that ACHD fellowship programs need to supply more information on their websites to provide applicants with details to help them choose the fellowship program that corresponds best with their career goals. Expanding upon information regarding recruitment, education, research, and incentives will provide applicants with a strong understanding of ACHD fellowship programs and what they can expect throughout their education. In return, this will help ACHD fellowship programs attract stronger applicants, ultimately improving the quality of their respective programs.

## Introduction

Adult Congenital Heart Disease (ACHD) fellowship is an option for physicians who have completed their internal medicine residency and cardiology fellowship. Prospective ACHD applicants rely on the websites of fellowship programs to make informed decisions regarding which programs to apply for [1]. Access to information regarding a fellowship program is crucial for prospective applicants as it helps them evaluate which programs best align with their career goals, objectives, and criteria for selecting a program. Previous research has shown that the online presence of residency programs was significant in an applicant's decision to apply to the respective program, with almost 80% of applicants stating that the online information about residency programs had influenced their decision [2]. Key factors such as curriculum, rotations, compensation, benefits, and research opportunities were consistently considered by applicants when choosing residency programs [3]. Applicants expect to find such information when they search online for fellowship programs. However, studies have shown that many residency and fellowship programs do not provide this information on their websites [2,4]. Considering the importance of this information to prospective applicants, fellowship programs must take steps to include these details on their websites to make them more appealing to applicants. Therefore, this study aims to provide ACHD fellowship websites with suggestions to include information that is inadequately addressed to inform future ACHD fellowship applicants.

Providing comprehensive details about fellowship programs is crucial in attracting qualified applicants. The absence of such information can lead to fewer potential candidates applying for the program. Therefore, it is essential that ACHD fellowship programs and other programs include information about their curriculum, rotations, compensation, benefits, and research opportunities on their websites. This data would provide prospective applicants with a clear idea of what to expect from the program and help them make an informed decision about their future careers.

## Materials and methods

The study analyzed 25 ACHD fellowship websites listed on the Fellowship Residency Electronic Interactive Database (FREIDA) in March 2023. The websites were evaluated by 34 criteria grouped into three categories of information: recruitment, education and research, and incentives. The 34 criteria were identified based on previous research evaluating website content for residency and fellowship programs self-reported by applicants [3,5,6-9]. The websites were accessed via the FREIDA link. If the link was broken, the ACHD programs were searched on Google.

The authors browsed through the respective ACHD fellowship website or description on FREIDA and looked for each of the 34 criteria. If the criteria were addressed on the given fellowship website or FREIDA, it was marked as addressed. The authors did not reach out to any fellowship directors, fellowship faculty, or current fellows for information; only the ACHD fellowship websites and FREIDA were used. If the criteria were found on third-party websites besides the websites of the ACHD fellowship program or FREIDA, it was marked as not addressed.

## Results

**Figure 1:**
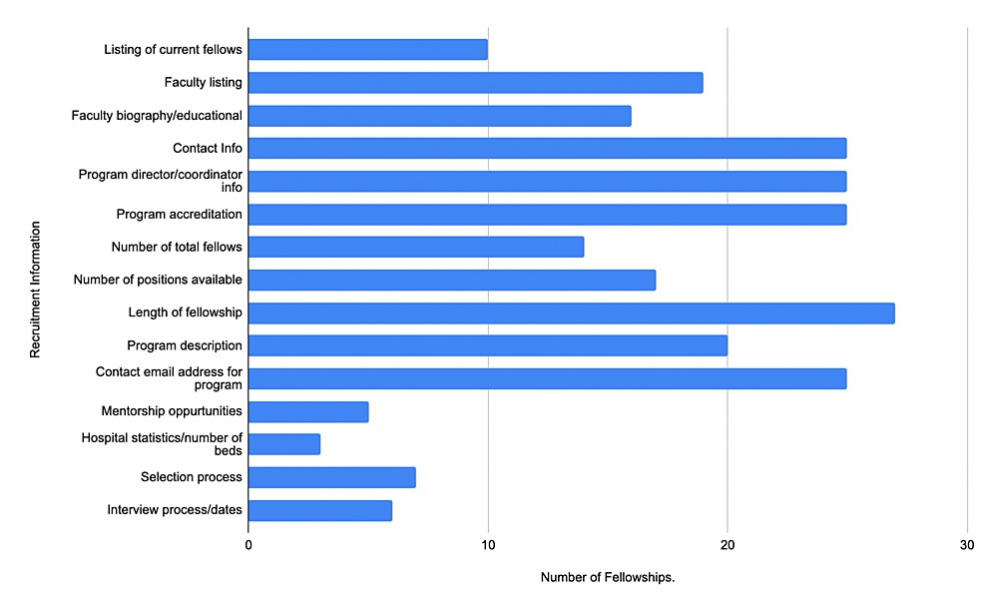
Recruitment information of ACHD fellowship websites ACHD: Adult Congenital Heart Disease

At the time of analysis, in March 2023, 25 ACHD fellowship programs were identified via FREIDA [10]. The ACHD fellowship programs that were included are the University of California Los Angeles, the University of California San Francisco, Stanford University, the University of Colorado, Emory University, Indiana University, Brigham and Women's Hospital, the University of Michigan, Mayo Clinic, University of Washington, University of Mississippi, Duke University, University of Nebraska, Icahn School of Medicine, New York University, New York Presbyterian Hospital, University of Cincinnati, Ohio State University, Oregon Health and Science University, University of Pennsylvania, Medical University of South Carolina, Vanderbilt University, Baylor University, University of Utah, Washington University, University of Wisconsin, and Medical College of Wisconsin. An analysis of ACHD fellowship websites' recruitment information showed that all fellowship websites provided information on contact information, program director/coordinator information, program accreditation, and contact email addresses for programs (Figure 1). On the other hand, most of the fellowship programs did not provide information about the listing of current fellows, mentorship opportunities, hospital statistics, selection process, and interview process/dates (Figure 1).

**Figure 2:**
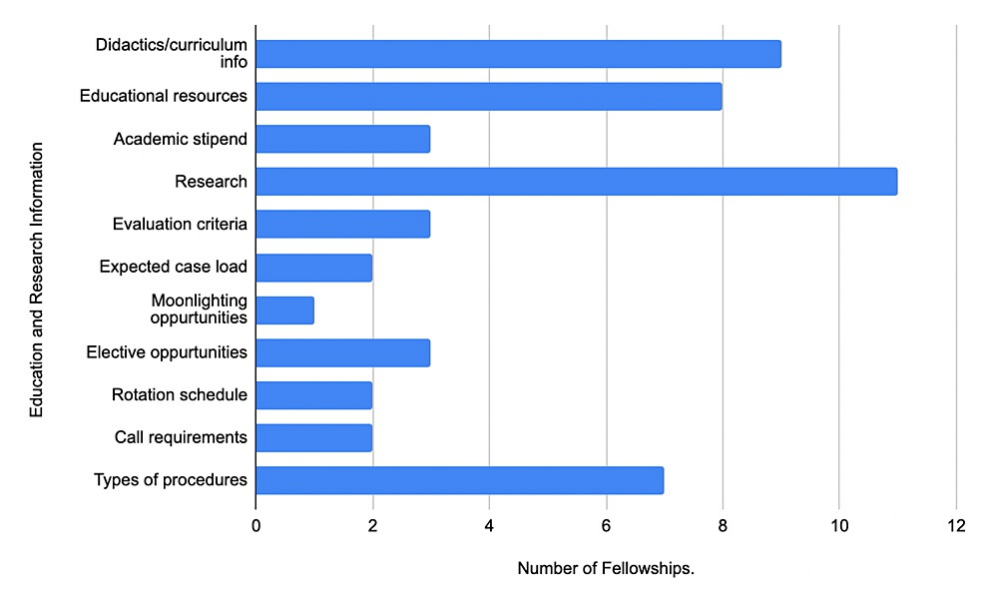
Education and research information of ACHD fellowship websites ACHD: Adult Congenital Heart Disease

**Figure 3:**
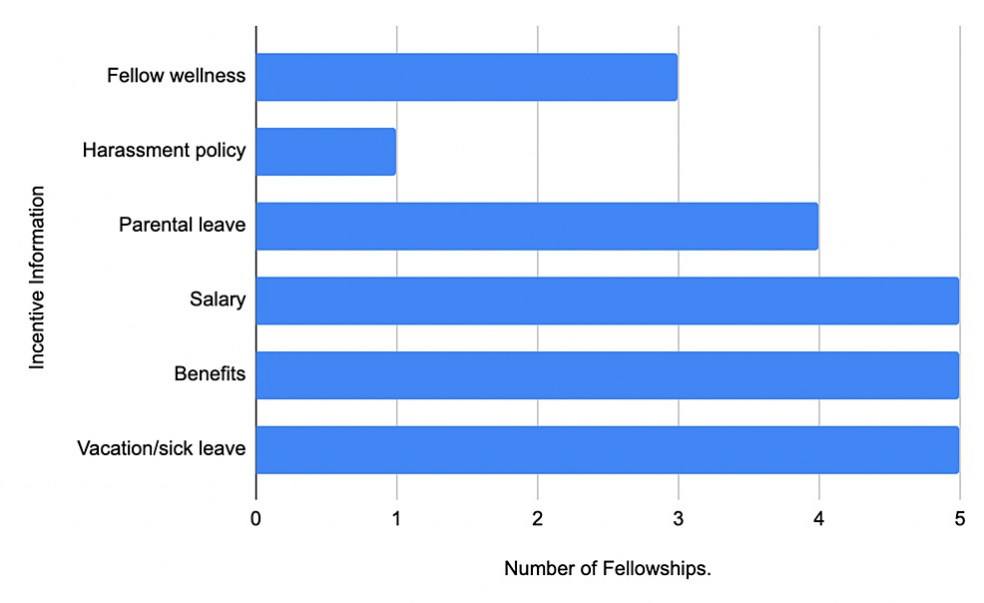
Incentive information of ACHD fellowship websites ACHD: Adult Congenital Heart Disease

In the education and research category, the most accessible category on ACHD fellowship websites was research information at 44% (11/25), with didactics/curriculum information coming second at 36% (9/25) and educational resources coming third at 32% (8/25) (Figure 2). Only one ACHD fellowship website provided information on moonlighting opportunities (Figure 2). With respect to the incentive information category, only 20% (5/25) of ACHD fellowship websites provided information on salary, benefits, and vacation/sick leave (Figure 3). Only one ACHD fellowship website provided information on their harassment policy (Figure 3).

In regards to the evaluation of ACHD fellowship websites, ACHD fellowship websites can be improved in multiple ways in the criteria that applicants seek when applying for fellowships (Table 1). Researchers identified a majority of these criteria were under 50%, which included the listing of current fellows, mentorship opportunities, hospital statistics, selection process, interview process/dates, education/research information, and incentive information (Table 1).

**Table 1:** Website information on Adult Congenital Heart Disease Fellowship Programs, n=25

Evaluation criteria	Programs addressing criteria	Percentage
Listing of current fellows	10	37%
Faculty listing	19	70%
Faculty biography/educational	16	59%
Contact info	26	96%
Program director/coordinator info	26	96%
Program accreditation	25	93%
Number of total fellows	14	52%
Number of positions available	17	63%
Length of fellowship	27	100%
Program description	20	74%
Contact email address for program	26	96%
Mentorship opportunities	5	19%
Hospital statistics/number of beds	3	11%
Selection process	7	26%
Interview process/dates	6	22%
Didactics/curriculum info	9	33%
Educational resources	8	30%
Academic stipend	3	11%
Research	11	41%
Evaluation criteria	3	11%
Expected case load	2	7%
Moonlighting opportunities	1	4%
Elective opportunities	3	11%
Rotation schedule/work	2	7%
Call requirements/schedule	2	7%
Types of procedures	7	26%
Fellow wellness	3	11%
Harassment policy	1	4%
Parental leave	4	15%
Salary	5	19%
Benefits	5	19%
Vacation/sick leave	5	19%

## Discussion

This study of ACHD fellowship websites and their accessibility to information uncovered significant areas that need improvement to provide sufficient information for applicants to ensure they have adequate details of fellowship programs they wish to apply to. A significant number of ACHD fellowship websites lacked information on recruitment information, education and research opportunities, and incentive information. Without these details, applicants will lack critical information that can help them compare fellowship programs. Prospective fellows will struggle to fully grasp what each program can deliver or what is anticipated of them as a fellow [3]. 

Regarding recruitment information, a majority of ACHD fellowship programs lacked details in listing current fellows, the number of total fellows that have graduated from their program, mentorship opportunities, hospital statistics, the selection process, and the interview process. Recruitment details like these criteria can provide applicants with important communication, expectations during the application process, and opportunities to contact past and current fellows to gather insight about the respective fellowship program [11]. 

Most ACHD fellowship programs did not provide much information regarding education and research information. Didactic information, educational resources, academic stipend, evaluation criteria, expected caseload, moonlighting opportunities, elective opportunities, rotation schedule, call requirements, and types of procedures were addressed by 35% or less of ACHD fellowship websites. These details provide applicants with aspects of the fellowship program that they should expect if accepted. If applicants are accepted to programs that have insufficient information regarding their education and research, it can lead to the applicant's expectations not being met concerning the program [12]. Description of educational and research resources on ACHD fellowship websites will help applicants make more informed decisions on which ACHD fellowship program to select [13].

Lastly, incentive information appeared to be seldom addressed by ACHD fellowship programs. Fellow wellness, harassment policy, parental leave, salary, benefits, and vacation/sick leave was addressed by 20% or less by ACHD fellowship websites. If applicants join these programs, they may not have the knowledge or opportunity to address incidents or stressors that may impact their wellness and education. Sufficient details of incentives can encourage applicants to apply to these programs with the assurance that they will have opportunities to address other responsibilities in their life, take vacations, or report harassment, overall translating to an increase in fellow wellness [14].

This study, however, has its limitations. Since the analysis of ACHD fellowship websites in March 2023, ACHD fellowship programs may have updated their websites to include more details about their respective programs. Additionally, third-party websites, which were excluded from this study, have the possibility to pose important information to applicants about fellowship programs that may not have been discussed on FREIDA or the respective fellowship website [15].

## Conclusions

A vast majority of ACHD fellowship websites can provide more information to applicants regarding their program. This research's objective is to supply fellowship websites with suggestions to improve their websites to help bring more applicants to their program. Key details such as recruitment, education, research, and incentive information can provide applicants with holistic knowledge and what to expect from these ACHD fellowship programs. By providing accessibility to information, applicants are well-equipped to make informed decisions on which fellowship programs they would like to apply to that best serve their interests and expectations. Through fulfilling these expectations, applicants, once accepted to these programs, will better understand what is expected of them, as well as what they expect from the program, leading to a fulfilling fellowship experience.

## References

[1] Yan Q, Jensen K, Field A (2021). Critical evaluation of the efficiency of colorectal Fellowship websites: cross-sectional study. JMIR Med Educ.

[2] Chen VW, Hoang D, Garner W (2018). Do websites provide what applicants need? Plastic surgery residency program websites versus applicant self-reported needs. Plast Reconstr Surg Glob Open.

[3] Gaeta TJ, Birkhahn RH, Lamont D, Banga N, Bove JJ (2005). Aspects of residency programs' web sites important to student applicants. Acad Emerg Med.

[4] Kirkendoll SD, Carmody JB, Rhone ET (2021). Information quality for residency applicants in fellowship and Residency Electronic Interactive Database (FREIDA) and program websites. Cureus.

[5] Phitayakorn R, Macklin EA, Goldsmith J, Weinstein DF (2015). Applicants' self-reported priorities in selecting a residency program. J Grad Med Educ.

[6] Lu F, Vijayasarathi A, Murray N, Hamid S, Khosa F (2021). Evaluation of pediatric radiology fellowship website content in USA and Canada. Curr Probl Diagn Radiol.

[7] Chu LF, Young CA, Zamora AK, Lowe D, Hoang DB, Pearl RG, Macario A (2011). Self-reported information needs of anesthesia residency applicants and analysis of applicant-related web sites resources at 131 United States training programs. Anesth Analg.

[8] Raza SS, Asban A, Donahue J, Wei B (2022). Analysis of applicants' perspectives of cardiothoracic surgery fellowship program websites. Ann Thorac Surg.

[9] Young BL, Cantrell CK, Patt JC, Ponce BA (2018). Accessibility and content of individualized adult reconstructive hip and knee/musculoskeletal oncology fellowship web sites. Arthroplast Today.

[10] (2023). Fellowship and residency electronic interactive database (FREIDA) online. https://freida.ama-assn.org/.

[11] Stoeger SM, Freeman H, Bitter B, Helmer SD, Reyes J, Vincent KB (2019). Evaluation of general surgery residency program websites. Am J Surg.

[12] Ahmed F, Ali B, Haque MZ, Mohammed I, Bazzy Y (2023). Cross-sectional content evaluation of spinal cord injury Medicine Fellowship websites. Cureus.

[13] Mulcahey MK, Gosselin MM, Fadale PD (2013). Evaluation of the content and accessibility of web sites for accredited orthopaedic sports medicine fellowships. J Bone Joint Surg Am.

[14] Wong TY, Huang JJ, Hoffmann JC, Flug JA, Cooke EA, Donnelly EF (2022). Resident wellness in radiology as portrayed by departmental websites. Acad Radiol.

[15] Kutikov A, Morgan TM, Resnick MJ (2009). The impact of residency match information disseminated by a third-party website. J Surg Educ.

